# Animal models for clinical and gestational diabetes: maternal and fetal outcomes

**DOI:** 10.1186/1758-5996-1-21

**Published:** 2009-10-19

**Authors:** Ana CI Kiss, Paula HO Lima, Yuri K Sinzato, Mariana Takaku, Marisa A Takeno, Marilza VC Rudge, Débora C Damasceno

**Affiliations:** 1 Laboratory of Experimental Research of Gynecology and Obstetrics, Department of Gynecology and Obstetrics, Botucatu Medical School - São Paulo State University (Unesp), Botucatu, São Paulo, Brazil

## Abstract

**Background:**

Diabetes in pregnant women is associated with an increased risk of maternal and neonatal morbidity and remains a significant medical challenge. Diabetes during pregnancy may be divided into clinical diabetes and gestational diabetes. Experimental models are developed with the purpose of enhancing understanding of the pathophysiological mechanisms of diseases that affect humans. With regard to diabetes in pregnancy, experimental findings from models will lead to the development of treatment strategies to maintain a normal metabolic intrauterine milieu, improving perinatal development by preventing fetal growth restriction or macrosomia. Based on animal models of diabetes during pregnancy previously reported in the medical literature, the present study aimed to compare the impact of streptozotocin-induced severe (glycemia >300 mg/dl) and mild diabetes (glycemia between 120 and 300 mg/dl) on glycemia and maternal reproductive and fetal outcomes of *Wistar *rats to evaluate whether the animal model reproduces the maternal and perinatal results of clinical and gestational diabetes in humans.

**Methods:**

On day 5 of life, 96 female *Wistar *rats were assigned to three experimental groups: control (n = 16), severe (n = 50) and mild diabetes (n = 30). At day 90 of life, rats were mated. On day 21 of pregnancy, rats were killed and their uterine horns were exposed to count implantation and fetus numbers to determine pre- and post-implantation loss rates. The fetuses were classified according to their birth weight.

**Results:**

Severe and mild diabetic dams showed different glycemic responses during pregnancy, impairing fetal glycemia and weight, confirming that maternal glycemia is directly associated with fetal development. Newborns from severe diabetic mothers presented growth restriction, but mild diabetic mothers were not associated with an increased rate of macrosomic fetuses.

**Conclusion:**

Experimental models of severe diabetes during pregnancy reproduced maternal and fetal outcomes of pregnant women presenting uncontrolled clinical diabetes. On the other hand, the mild diabetes model caused mild hyperglycemia during pregnancy, although it was not enough to reproduce the increased rate of macrosomic fetuses seen in women with gestational diabetes.

## Background

Diabetes mellitus (DM) is a disease characterized by disarrangements in carbohydrate, protein and lipid metabolism caused by the complete or relative insufficiency of insulin secretion and/or insulin action [1]. Diabetes in pregnant women is associated with an increased risk of maternal and neonatal morbidity and remains a significant medical challenge. Diabetes during pregnancy may be divided into clinical diabetes (women previously diagnosed with type 1 or type 2 diabetes) and gestational diabetes, defined as any glucose intolerance detected during pregnancy that has evolved from a diagnosis associated with the metabolic risk of type 2 diabetes to a clinical condition associated with higher risks for maternal and perinatal morbidity [2]. Fortunately, the prognosis has changed dramatically due to an increased clinical awareness of the potential risks for the mother and the infant.

Experimental models are developed with the purpose of enhancing understanding of the pathophysiological mechanisms of diseases that affect humans. With regard to diabetes in pregnancy, experimental findings from models will lead to the development of treatment strategies to maintain the closest to normal metabolic intrauterine milieu, improving perinatal development by preventing fetal growth restriction or macrosomia. The rat (and the rabbit) is often used in reproductive toxicity studies [3]. In general, the uncontrolled human type 1 DM clinical status during pregnancy is reproduced by streptozotocin (STZ) administration (40 mg/kg) to rats during adult life using the venous route [4-6]. In this experimental model, rats present with severe diabetes, with glycemia above 300 mg/dl, and the fetuses of dams are classified as small fetuses for gestational age, characterizing intrauterine growth restriction. Human type 2 DM and gestational DM conditions are reproduced in animals by administration of different doses of STZ in the neonatal period [7-16], before mating [17-20] or during pregnancy [21-30]. Adult animals present with glycemia between 120 and 300 mg/dl, characterizing moderate or mild diabetes [31-33]. Merzouk and colleagues [23-25] and Soulimane-Mokhtari and colleagues [30] verified that mildly hyperglycemic dams have fetuses that are large for gestational age, classified as macrosomic.

Evidence in the literature indicates that neonatal rats treated with STZ at birth exhibit altered insulin and glucose tolerance tests [8,9,13] and plasmatic insulin [11,15]. Based on the insulin action response and glucose tolerance test, Triadou and colleagues [15] established an experimental design that reproduces the development of gestational diabetes in women. Several reports in the literature describe the effects of severe and mild diabetes on pregnancy, fetal glycemia and development, but these studies did not investigate correlations between maternal and fetal repercussions in these two different glycemic ranges. Therefore, the present study aimed to compare the impact of STZ-induced severe and mild diabetes on glycemia and maternal reproductive and fetal outcomes of *Wistar *rats to evaluate whether the animal model reproduces the maternal and perinatal results of clinical and gestational diabetes in humans.

## Methods

### Subjects

*Wistar *rats were obtained from São Paulo State University (Unesp) Botucatu, São Paulo State, Brazil. They were maintained in an experimental room under controlled conditions of temperature (22 ± 2°C), humidity (50 ± 10%), and a 12-hour light/dark cycle. All experimental procedures presented in this study were approved by the local Committee of Ethics in Animal Experimentation, which assures adherence to the standards established by the Guide for the Care and Use of Laboratory Animals.

### Experimental procedures

On day 5 of life, 64 female *Wistar *rats were randomly assigned to three experimental groups: control (n = 16) - rats that received citrate buffer solution (0.1 M, pH 6.5) intraperitoneally on day 5 of life; severe diabetes (n = 50) - rats that received STZ (SIGMA Chemical Company, St Louis, MO, USA; 40 mg/kg intravenously) on day 75 of life; and mild diabetes (n = 30) - rats that received STZ (70 mg/kg intraperitoneally) dissolved in citrate buffer solution on day 5 of life. At day 90 of life, the female rats were mated and the morning on which sperm were found in the vaginal smear was designated pregnancy day 0. Blood samples were obtained from cut tail tips for glycemic determination (glucose oxidase) using a typical glucometer; values are expressed in milligrams per deciliter (mg/dl). Inclusion criteria required glycemia levels on day 0 of pregnancy of <120 mg/dl for the control group (n = 16), >300 mg/dl for the severe diabetic group (n = 18), and between 120 and 300 mg/dl for the mild diabetic group (n = 6). Body weight, food intake and glycemia were evaluated on days 0, 7, 14 and 21 of pregnancy. On day 21 of pregnancy, the dams were anesthetized with sodium pentobarbital (Hypnol^® ^3%) and their uterine horns exposed to count implantation and fetus numbers for determination of pre- and post-implantation loss rates. The fetuses were removed, weighed and classified according to their birth weight as follows: large for pregnancy age (LPA) if their birth weight was greater than 1.0 standard deviation of the mean birth weight of the control dam pups; small for pregnancy age (SPA) if their birth weight was lower than 1.0 standard deviation of the mean birth weight of the control dam pups; and appropriate for pregnancy age (APA) if their birth weight was included in ± 1.0 standard deviation of the mean birth weight of the control dam pups [34]. Blood pool glycemia levels were determined from three newborns from each litter.

### Statistical analysis

Results are presented as mean ± standard error of mean. The proportion test (Chi-square) was used for fetal weight classification. Two-way analysis of variance (ANOVA) followed by the Student-Newman-Keuls test was employed to compare the data for maternal glycemia, food intake, body weight during pregnancy, number of implantation sites and number of live fetuses. Pre- and post-implantation loss rates were analyzed by Mann Whitney non-parametric test. Maternal and fetal glycemia correlation was determined using Pearson correlation. The statistical significance interval is considered as *P *< 0.05 for all data. All statistical analyses were performed with Statistica software (Statsoft, Tulsa, OK, USA).

## Results

All 16 rats assigned to the control group were mated, had a positive pregnancy diagnosis and were included in this study. Only 18 of 50 rats administered STZ as adults (severe diabetic rats) had a positive pregnancy diagnosis and were included in this study following the inclusion criteria for their experimental group. All 30 rats administered streptozotocin as neonates were also mated, but only 16 presented with a positive pregnancy diagnosis and only 6 achieved the inclusion criteria. The rats that did not reach inclusion criteria were used in another study. There were no significant differences in the number of implantation sites in the severe and mild diabetic groups compared to the control group nor between the severe and mild diabetic groups. A lower mean number of live fetuses and a higher post-implantation loss rate were observed in severe diabetic rats compared to the control and mild diabetes groups (Table 1).

**Table 1:** Maternal reproductive outcomes of control, severe diabetic and mild diabetic rats

**Variables**	**Control**	**Severe diabetes**	**Mild diabetes**
Number of rats used	16	50	30
Number of rats that achieved inclusion criteria	16 (100%)	18 (36%)	6 (20%)
Implantation number	159	199	70
Mean ± SEM	11.67 ± 0.33	11.71 ± 0.39	11.67 ± 0.56
Live fetus number	153	115	66
Mean ± SEM	11.50 ± 0.22	6.76 ± 1.15^a, b^	11.00 ± 0.37
Pre-implantation loss (%)	4.85%	8,44%	1,52%
Post-implantation loss (%)	1.28%	42,27%^c, d^	5,24%

Rats were injected with citrate buffer solution (control), streptozotocin as adults (severe diabetes) and streptozotocin during the neonatal period (mild diabetes). Values are presented as mean ± standard deviation and proportions (%). ^a^*P *< 0.05 - statistically significant difference compared to control group (Student Newman Keuls); ^b^*P *< 0.05 - statistically significant difference compared to mild diabetes group (Student Newman Keuls); ^c^*P *< 0.05 - statistically significant difference compared to control group (Mann Whitney); ^d^*P *< 0.05 - statistically significant difference compared to mild diabetes group (Mann Whitney). SEM, standard error of the mean.

**Figure 1:**
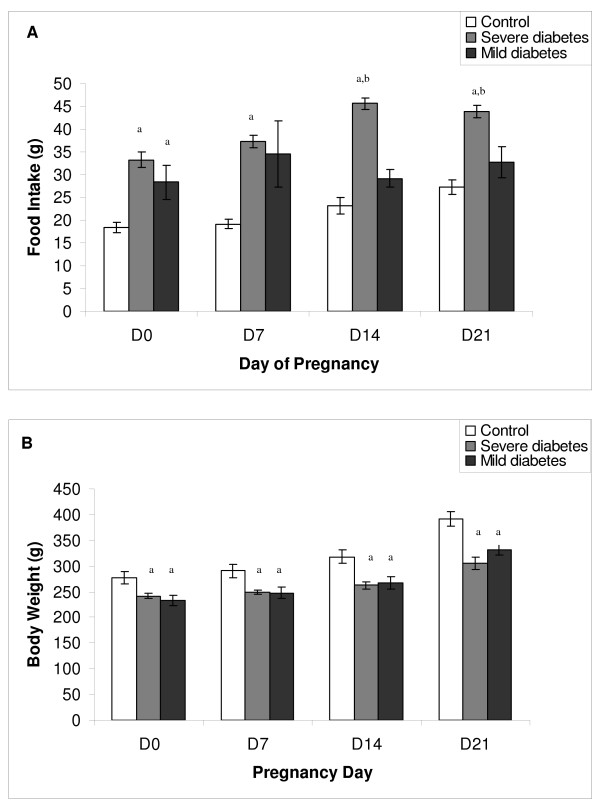
**Effect of streptozotocin induced diabetes on food intake and body weight of rats during pregnancy**. (A) Food intake and (B) body weight on days 0, 7, 14 and 21 of pregnancy of rats injected with citrate buffer solution (control), streptozotocin as adults (severe diabetes) and streptozotocin during the neonatal period (mild diabetes). Values are presented as mean ± standard error of mean. ^a^*P *< 0.05 - statistically significant difference compared to control group (Student Newman Keuls); ^b^*P *< 0.05 - statistically significant difference compared to the mild diabetes group (Student Newman Keuls).

Rats with severe diabetes had a higher food intake compared to mild diabetic rats on days 14 to 21 of pregnancy, and compared to control rats on all days of pregnancy. Mild diabetic rats had a higher food intake compared to the control group only on day 0 of pregnancy (Figure 1A). Both severe and mild diabetic rats had lower body weight compared to the control group (Figure 1B).

During their entire pregnancy, control rats had normal glycemic values (around 80 mg/dl). Glycemia remained above 300 mg/dl in the severe diabetic rats and between 120 and 300 mg/dl in the mild diabetic rats. Both severe and mild diabetic rats had higher glycemia levels throughout pregnancy compared to the control group. When compared to the mild diabetes group, severe diabetic dams had higher glycemia levels prior to mating and during pregnancy. Newborns from severe diabetic dams had higher glycemia levels compared to newborns from both the control and mild diabetic groups (Table 2). There was a positive correlation (*P *< 0.05) between maternal and fetal glycemia in all experimental groups.

**Table 2:** Glycemia of control, severe diabetic and mild diabetic rats throughout pregnancy and of newborns

	**Control (n = 16)**	**Severe diabetes (n = 18)**	**Mild diabetes (n = 6)**
Prior mating	84.33 ± 0.76	343.56 ± 14,36^a, b^	177.12 ± 45.53
Day 0	78.17 ± 3.89	351.78 ± 12.79^a, b^	186.67 ± 26.04^a^
Day 7	77.67 ± 3.68	294.11 ± 11.01^a, b^	177.67 ± 32.84^a^
Day 14	77.17 ± 5.85	327.44 ± 12.70^a, b^	179.69 ± 39.85^a^
Day 21	79.83 ± 6.65	322.61 ± 17.95^a, b^	170.67 ± 30.21^a^
Newborns	68.25 ± 7.94	464.33 ± 28.95^a, b^	115.9 ± 37.57

Glycemia (mean ± standard error of mean) were taken prior to mating and on days 0, 7, 14 and 21 of pregnancy from rats injected with citrate buffer solution (control), streptozotocin as adults (severe diabetes) and streptozotocin during the neonatal period (mild diabetes). Blood pool glycemia was determined from three newborns from each litter. ^a^*P *< 0.05 - statistically significant difference compared to control group (Student Newman Keuls);

^b^*P *< 0.05 - statistically significant difference compared to mild diabetes group (Student Newman Keuls).

In both the severe and mild diabetes groups, there was a higher proportion of SPA fetuses and a reduced percentage of APA and LPA fetuses compared to the control group. Severe diabetic rats also had higher SPA and lower APA rates compared to mild diabetic rats. The proportions of LPA fetuses from the severe and mild diabetes groups were similar (Table 3).

**Table 3:** Fetal weight classification of offspring born to control, severe diabetic and mild diabetic rats

**Variable/groups**	**Control**	**Severe diabetes**	**Mild diabetes**
SPA	46/192 (24%)	112/129 (87%)^a, b^	39/65 (60%)^a^
APA	99/192 (52%)	14/129 (11%)^a, b^	22/65 (34%)^a^
LPA	47/192 (24%)	3/129 (2%)^a^	4/65 (6%)^a^

Fetal weight classification is defined as small for pregnancy age (SPA), appropriate for pregnancy age (APA) or large for pregnancy age (LPA). The offspring were born to rats injected with citrate buffer solution (control), streptozotocin as adults (severe diabetes) or streptozotocin during the neonatal period (mild diabetes). Values are number/total (percent). ^a^*P *< 0.05 - statistically significant difference compared to control group (Chi-square test); ^b^*P *< 0.05 - statistically significant difference compared to mild diabetes group (Chi-square test).

## Discussion

STZ is often used to induce DM in experimental animals due to its toxic effects on pancreatic beta-cells [35,36]. It is a potent alkylating agent able to methylate DNA [37-39] and although it is generally accepted that the cytotoxicity produced by STZ depends on DNA alkylation [37,39], several lines of evidence indicate that free radicals play an essential role in its mechanism of DNA damage and cytotoxicity. The nitrosurea moiety of STZ is responsible for its cellular toxicity, which is probably mediated through a decrease in NAD levels and the production of intracellular free radicals. The deoxyglucose moiety of STZ facilitates its transport across the cell membrane, in which the GLUT-2 glucose-transporter appears to play an essential role. The insulin-producing beta-cells of the islets of Langerhans not only express high levels of GLUT-2 transporters but also have a relatively low NAD content, making them particularly vulnerable to STZ toxicity [40].

In the mild diabetes group, STZ treatment created a range of damage to beta cells, leading to a variable range of insulin insufficiency. Only 6 (20%) of the initial 30 rats had a positive pregnancy diagnosis and presented with mild diabetes on pregnancy day 0 according to the inclusion criteria previously established (glycemia between 120 and 300 mg/dl). Although the success rate of this model may appear low, models in which high doses of STZ are administered in the neonatal period to achieve mild diabetes are well established [7-16]. However, these studies do not mention how many animals achieved hyperglycemia in adult life. STZ has a beta-cell specific toxicity that produces severe and permanent diabetes when given to adult rats. When given during the neonatal period, there is a spontaneous recovery from the damage caused to the beta-cells in the first 2 weeks of life. However, beta-cell regeneration is incomplete and this reduced beta-cell mass results in the appearance of a form of diabetes in adult life that resembles DM type 2 in humans [9]. Individual differences in STZ metabolism [41] and beta-cell regeneration capacity [9] may explain why so many rats that receive STZ do not present mild diabetes in adult life.

In the present study, rats with glycemia above 300 mg/dl (severe diabetes) had higher food intake but reduced body weight during pregnancy, both common features of the severe diabetic state. The reduced body weight is a consequence of metabolic alterations caused by hyperglycemia/hypoinsulinemia, such as asthenia, as described by Damasceno and colleagues [4]. Rats injected neonatally with STZ had mild diabetes (glycemia from 120 to 300 mg/dl) without a significant increase in food intake, but reduced body weight, which can also be explained by metabolic alterations despite the lower glycemia compared to the severely diabetic rats. Although maternal hypoinsulinemia/hyperglycemia has a major impact on fetal weight, the reduced maternal body weight of mild diabetic rats, resulting from low weight gain during pregnancy, could be a cause of the low number of LPA fetuses in this group.

Severe diabetic rats had glycemia levels above 300 mg/dl throughout pregnancy. This result was expected and is in agreement with other studies previously performed in our laboratory [4-6], reproducing the hyperglycemia that some women with uncontrolled clinical diabetes present during pregnancy. The mild diabetic rats maintained their glycemia between 120 and 300 mg/dl during pregnancy. STZ administration in the neonatal period caused mild hyperglycemia during pregnancy, which has also been reported by Triadou and colleagues [15], Capobianco and colleagues [10] and Kiss [42], reproducing the hyperglycemia that some women with gestational diabetes present during pregnancy.

In our study, the lower number of live fetuses and the high post-implantation loss rate in the severe diabetes group are characteristic of a hyperglycemic (glycemia above 300 mg/dl) intrauterine milieu, and are in agreement with other studies [6,43]. In the present study, the high glycemic levels did not prevent embryo implantation but did impair development, leading to fetal death, as confirmed by the low number of live fetuses. Our results also show that rats with severe diabetes had newborns with intrauterine growth restriction. This can be explained by fetal beta-cell collapse, which eventually leads to fetal hypoinsulinemia that causes the growth restriction [19,44,45].

There is evidence that the hyperglycemic intrauterine milieu of a mildly diabetic mother stimulates the fetal endocrine pancreas to hyperinsulinemia and accelerated anabolism, resulting in fetal and neonatal macrosomia. Many reports in the literature indicate that animal models in which STZ is injected during the neonatal period are compatible with human gestational diabetes conditions, with the presence of macrosomic fetuses [23-25,30] that are intolerant to glucose [44,46]. In contrast, our results show that the mild diabetic dams did not have an increased percentage of newborns classified as LPA. Similarly, Kervran and colleagues [19] also did not obtain macrosomic fetuses when studying the offspring of rats with mild hyperglycemia during pregnancy, and suggest that the differences between the clinical findings in humans and the experimental results using rats are due to the short pregnancy time in the rat and differences in the percentages of adipose tissue in rat fetuses (1%) and human offspring (16%) and the greater weight gain in the human species.

The offspring of the mild diabetic dams did not have impaired glycemia compared to the control group. However, the offspring of the severe diabetic dams showed higher glycemia levels compared to both the control and mild diabetes groups. Many clinical and experimental studies have shown that offspring that developed in an intrauterine milieu that has been modified by hyperglycemia show intolerance to glucose [44,46]. In the present study, offspring were not submitted to the glucose tolerance test, so there is no evidence that they are intolerant to glucose, but their glycemia levels correlate positively with those of their mothers. Kervran and colleagues [19] also observed a positive correlation between maternal and fetal glycemia levels in both severe and mild diabetic dams.

## Conclusion

STZ-induced severe and mild diabetic dams showed different glycemic responses during pregnancy, although both adversely affected fetal glycemia and weight, confirming that maternal glycemia is directly associated with fetal development. Newborn from severe diabetic mothers presented intrauterine growth restriction, but mild diabetic mothers did not have an increased percentage of LPA fetuses. The experimental model of severe diabetes during pregnancy reproduced maternal and fetal outcomes of women with uncontrolled clinical diabetes. On the other hand, the mild diabetes model caused mild hyperglycemia during pregnancy, although it was not enough to reproduce the increased rate of macrosomic fetuses seen in women with gestational diabetes.

## Abbreviations

APA: appropriate for pregnancy age; DM: diabetes mellitus; LPA: large for pregnancy age; SPA: small for pregnancy age; STZ: streptozotocin.

## Competing interests

The authors declare that they have no competing interests.

## Authors' contributions

ACIK participated in the acquisition, analysis and interpretation of data and helped to draft the manuscript. PHOL participated in the acquisition of data and helped to draft the manuscript. YKS participated in the acquisition of data and helped to draft the manuscript. MT participated in the acquisition of data and helped to draft the manuscript. MAT participated in the acquisition of data and helped to draft the manuscript. MVCR helped to draft the manuscript. DCD conceived the study, participated in its design, coordination, analysis and interpretation of data and helped to draft the manuscript. All authors read and approved the final manuscript.
